# Polymerization Shrinkage Evaluation of Restorative Resin-Based Composites Using Fiber Bragg Grating Sensors

**DOI:** 10.3390/polym11050859

**Published:** 2019-05-11

**Authors:** Rodrigo Lins, Alexandra Vinagre, Nélia Alberto, Maria F. Domingues, Ana Messias, Luís R. Martins, Rogério Nogueira, João C. Ramos

**Affiliations:** 1 Department of Restorative Dentistry, Piracicaba Dental School, University of Campinas, Av. Limeira, 901, Areião, Piracicaba–SP 13414-903, Brazil; rodrigowlins@hotmail.com (R.L.); martins@unicamp.br (L.R.M.); 2 Institute of Operative Dentistry, Dentistry Area, Faculty of Medicine, University of Coimbra, Avenida Bissaya Barreto, Blocos de Celas, 3700-075 Coimbra, Portugal; ana.messias@uc.pt (A.M.); joaoctramos@sapo.pt (J.C.R.); 3 Instituto de Telecomunicações, Campus Universitário de Santiago, 3810-193 Aveiro, Portugal; nelia@av.it.pt (N.A.); fdomingues@av.it.pt (M.F.D.); rnogueira@av.it.pt (R.N.); 4 Instituto Português de Medicina Dentária, Rua José Luciano Castro, 141, Esgueira, 3800-207 Aveiro, Portugal

**Keywords:** resin based-composites, bulk-fill composite resins, light curing, polymerization shrinkage, optical fiber sensors, fiber Bragg gratings

## Abstract

The purpose of this study was to compare the linear polymerization shrinkage of different restorative resin-based composites (RBCs) using fiber Bragg grating (FBG) sensors. Five RBCs were evaluated: Zirconfill^®^ (ZFL); Aura Bulk-Fill (ABF); Tetric^®^ N-Ceram Bulk-Fill (TBF); Filtek^TM^ Bulk-Fill (FBF); and Admira Fusion-Ormocer^®^ (ADF). Ten samples per resin were produced in standardized custom-made half-gutter silicone molds. Two optical FBG sensors were used to assess temperature and polymerization shrinkage. Light curing was performed for 40 s and polymerization shrinkage was evaluated at 5, 10, 40, 60, 150, and 300 s. Statistical analysis was accomplished for normal distribution (Shapiro-Wilk, *p* > 0.05). Two-way repeated measures ANOVA with Greenhouse-Geisser correction followed by Bonferroni′s post-hoc test was used to analyze the linear shrinkage data (*p* < 0.05). ZFL showed the highest linear shrinkage and ADF the lowest. Shrinkage increased for all RBCs until 300 s, where significant differences were found between ADF and all other resins (*p* < 0.05). Among bulk-fill RBCs, TBF showed the lowest shrinkage value, but not statistically different from FBF. The ADF presented lower linear shrinkage than all other RBCs, and restorative bulk-fill composites exhibited an intermediate behavior.

## 1. Introduction

Resin-bases composites (RBCs) are the most used restorative materials for direct restoration procedures, both on anterior and posterior teeth. Since its development, a wide range of improvements has been made, both in the matrix and filler fraction, and also in initiator technology [1]. Although RBCs are considered materials with good physical and mechanical properties, they undergo a volumetric shrinkage between 2% to 5% during curing, proportional to the degree of monomer to polymer conversion [2]. This volumetric shrinkage induced by the polymerization is called intrinsic or total chemical shrinkage [3].

Resin composite polymerization comprises a pre- and post-gel phases. Before gel point, stress might be negligible because the material in pre-gel state can flow from the free surfaces to the bonded surface of restoration. After gelation, the formation of a semi-rigid polymer network hinders plastic deformation. Stress starts to develop if material is constrained by adhesion to cavity walls. When material accomplishes the vitrified state, its elastic modulus becomes higher and stress relaxation capacity diminishes significantly [2,3,4]. The resultant shrinkage stress can be transferred to the bonding interface and the remaining tooth structure, leading to interfacial adhesive failures, cuspal deflection, and/or enamel microcracks. These structural dysfunctions might impair or fully compromise the restoration. Besides, shrinkage stress is also largely determined by the visco-elastic properties of RBCs, defined by their flow capacity in early stages of the polymerization reaction, and elastic modulus development during polymer network formation. Which effect has the largest impact in shrinkage stress development is still controversial [1,5,6].

The attempt to reduce composite shrinkage strain remains an important goal in biomaterials research. Different strategies were developed for managing shrinkage stress of resin composites, including the use of low modulus intermediate liner materials acting as stress absorbers, incremental filling techniques, and alternative light application methods to delay curing kinetics [7]. Also, factors related to resin composite formulations, like changes in filler amount, shape or surface treatment, variations in monomer structure or chemistry and modification of initiator technology have been more recently introduced, aiming to reduce the polymerization shrinkage [5,6,8,9].

More recently, a novel class of RBCs, designated as low-shrinkage composites have emerged, which are generally allowed to be placed in a bulk fill mode. Bulk filling techniques are undoubtedly more user friendly than the meticulous incremental layering techniques required for conventional RBCs, allowing the placement of layers up to 5 mm, cured in a single-step.

Bulk fill composites were classified according to their rheological properties into base or full-body types. The base bulk-fill composites are flowable composites presenting low viscosity and lower filler content, generally requiring a surface capping with a conventional composite to accomplish mechanical and wear resistance to the restoration. The full-body bulk-fill composites have high filler loads, and are characterized as a paste-like highly viscous composite that can be used for the entire restoration, and are particularly indicated in high stress masticatory areas [10]. In vitro, the research strongly substantiates an increased depth of cure of bulk-fill resin composites over conventional composites, mainly attributed to their increased translucency [10]. Nevertheless, when comparing these materials, conflicting results regarding the development of contraction forces and shrinkage stress kinetics can be pointed out, with no clear advantage for all the bulk-fill ones [2,11,12]. A recent systematic review and meta-analysis showed that a similar clinical performance can be expected for bulk-fill and conventional resin composites in direct posterior restorations, encouraging the use of the former, as they reduce chair time and are less technically demanding [13].

Ormocer-based Bis-GMA-free resin composite materials have been more recently introduced for direct restorations. Ormocer is the acronym for organically modified ceramic and comprises inorganic-organic co-polymers with inorganic silanated filler particles. The solution and gelation (sol-gel) process form by hydrolysis and condensation of alkoxides, an inorganic Si-O-Si network characterized by a long inorganic silica chain backbone with organic lateral chains able to react during the curing process, using conventional photoinitiators [14]. Until now, only one brand of this recent generation of pure Ormocer composite resin, marketed as Admira Fusion-Ormocer^®^, is accessible. Scientific data is scarce concerning either in vitro or in vivo research [15,16,17]. Likewise, another restorative composite with diatomite fillers, marketed as Zirconfill^®^, was recently introduced, but there is no available scientific research with proper validation. 

Optical fiber sensors are currently being used in biomedical applications due to their reduced dimensions, chemical inertness, high sensitivity and resolution, compatibility, immunity to electromagnetic interference, possibility of real-time monitoring and the ease to be embedded in distinct materials [18]. These sensors have been used in the characterization of different dental materials, and these studies reveal the feasibility of this technology for this purpose, being highlighted the simplicity and the reliability to evaluate several characteristics of dental composites [19,20].

The aim of this study was to assess in real-time the linear shrinkage polymerization of five different RBCs, using FBG sensors. The null hypothesis was that there would be no differences in polymerization shrinkage behavior between the resin composites tested.

## 2. Fiber Bragg Gratings Theory

A FBG is a periodic modulation of the refractive index along the fibre core, which operates as a highly selective wavelength filter. When a FBG is illuminated by a broadband light source, only wavelengths that satisfy the Bragg condition are reflected, while all the others are transmitted [21]. The Bragg condition is given by:

(1) $$\lambda_{B}=2n_{eff}\Lambda$$

where *λ_B_* is the reflected Bragg wavelength, *n_eff_* is the effective refractive index of the fiber core, and Λ is the periodic modulation of the refractive index. The effective refractive index, as well as the periodic spacing between the grating planes, are affected by changes in strain (Δl) and/or temperature (Δ*T*). Consequently, the reflected Bragg wavelength changes, accordingly with the following equation:

(2) $$\Delta \lambda_{B}=\Delta \lambda_{B,\iota }+\Delta \lambda_{B,T}=2\left(\Lambda \frac{\partial n_{eff}}{\partial l}+n_{eff}\frac{\partial \Lambda }{\partial l}\right)\Delta l+2\left(\Lambda \frac{\partial n_{eff}}{\partial T}+n_{eff}\frac{\partial \Lambda }{\partial T}\right)\Delta T=S_{l}\Delta l+S_{T}\Delta T,$$

where the first term is the strain induced wavelength shift, and the last the thermal effect on the same parameter. *S_l_* and *S_T_* represent the strain and temperature sensitivity coefficients of the FBG sensors.

One drawback of the FBG based sensors is their cross-sensitivity to both strain and temperature variations. Several techniques have already been proposed in the literature to overcome this problem. Within this work, two FBG sensors were used in each experimental test, one of them inside a metallic needle, aiming to isolate the FBG from the resin, and consequently to respond only to temperature variations. The other one was in contact with the resin, being sensitive to both temperature and strain variations.

## 3. Materials and Methods

### 3.1. Dental Materials Samples

In this study, the linear polymerization shrinkage of five different RBCs was assessed: three full-body bulk-fill composites Aura Bulk-Fill; SDI (Bayswater, Victoria, Australia), Filtek^TM^ Bulk-Fill (3M ESPE, St Paul, Minnesota, USA) and Tetric^®^ N-Ceram Bulk-Fill (Ivoclar Vivadent AG, Schaan, Liechtenstein); one pure ormocer Admira Fusion-Ormocer^®^ (VOCO, Cuxhaven, Germany) and a nano-hybrid light-curable composite resin Zirconfill^®^ (Technew, Rio de Janeiro, Brazil). The product specifications are summarized in Table 1.

### 3.2. FBG Sensors

In the current study, FBGs with 1 mm length were inscribed into photosensitive single mode optical fiber (GF1, Thorlabs, Newton, NJ, EUA), with a UV light (248 nm) from a KrF excimer laser (BraggStar Industrial, LN, Santa Clara, CA, USA), using the phase mask technique. Before the inscription process, a small section of the acrylate fiber protection was removed in the FBG region, and later, no further recoating process was applied. The FBGs reflected spectra were monitored using the sm125-500 interrogation system (Micron Optics Inc., Atlanta, GA, USA), with a wavelength resolution of 1 pm and an acquisition frequency of 2 Hz, in the range of 1510 to 1590 nm.

After the FBG inscription process, the sensors were characterized to strain and temperature variations, aiming to determine the sensitivity coefficients. For the strain characterization, the optical fiber containing the FBG was fixed between a rigid fixed support and a linear translation stage, with a distance between anchorage points of 170 mm. The reflected Bragg wavelength was registered, as function of the imposed elongation, ranging from 0 to 0.48 mm, which corresponds to a maximum strain value of 2823.53 με. An average strain sensitivity coefficient of 0.0012 ± 0.0001 nm/με was obtained.

In the case of the temperature, a FBG and a FBG inside a 20Gx1” (Terumo Corp., Tokyo, Japan) metallic needle were thermally characterized using a climatic chamber (CH340, Angelantoni Industrie, Massa Martana, Italy). The temperature was increased from 15.0 to 85.0 °C, with step increments of 10.0 °C. The reflected Bragg wavelength was registered for each temperature level, after a stabilization period of 20 min, for the two FBGs. An average temperature sensitivity coefficient of 0.0093 ± 0.0002 nm/°C was obtained, for both sensors. Thus, the needle does not have influence on the thermal sensitivity of the FBG.

### 3.3. Specimen Preparation

**Figure 1:**
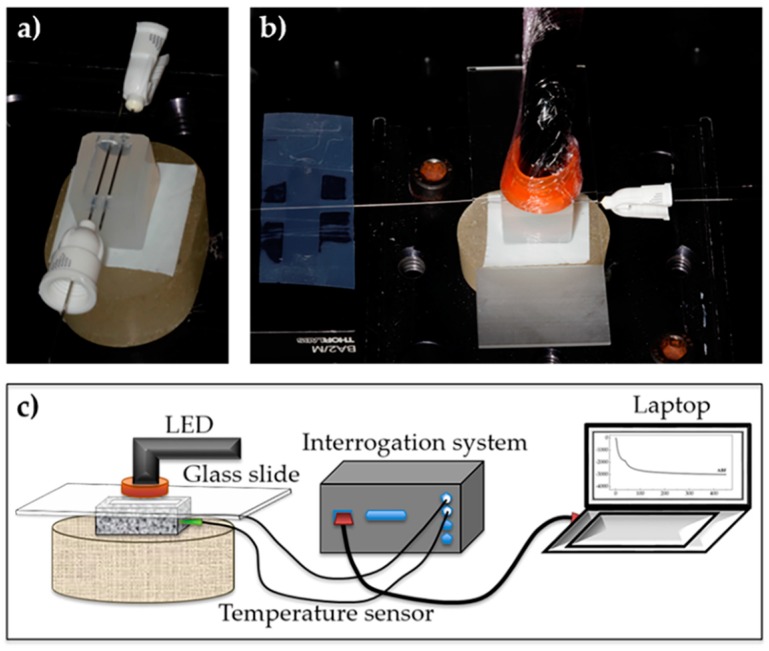
Photographs of the silicone mold with the two needles (**a**) and the light curing of the resin (**b**). Experimental setup used to assess the polymerization shrinkage of the RBCs (**c**).

Standardized custom-made half-gutter silicone molds with 6 mm depth; 9 mm wide and 15 mm length were prepared. Two metallic needles were positioned parallel 2 mm apart and longitudinal across the silicone mold, 2 mm below its superior limit (Figure 1a). Inside one of them was placed the FBG responsible for the temperature monitoring. In the other one, it was positioned the FBG for direct contact with the resin, to simultaneously measure temperature and deformation variations. The mold was filled with the uncured composite resin, and after this, the last needle was removed. This needle was only used to facilitate the insertion of the FBG through the mold silicone walls. The resin was then condensed in the mold, ensuring that the sensors were well wrapped in the material. Finally, the fiber tips of the FBG in direct contact with the resin were glued to two metallic supports and the FBG was slightly tensioned, causing a Bragg wavelength shift of about 0.5 nm, to allow a more accurate detection of the resin shrinkage, during the light curing process (Figure 1). This procedure has been previously used in these types of applications, and no adhesion loss of the FBG to the composite has been reported. Since the work′s aim is to assess the polymerization shrinkage of RBCs, this is nule (0 με) when the composite starts to be cured, so the Bragg wavelength registered at this moment, when the FBG is slightly tensioned, is considered to be the reference value, and the initial Bragg wavelength obtained after the FBG inscription process is ignored. To prevent oxygen inhibition and standardize light-curing tip distance, a 1 mm thick glass slide was positioned on the top of the silicone mold. For all tests, photo-activation was performed during 40 s, using a poly-wave LED light-curing unit (Bluephase N, Ivoclar Vivadent AG, Schaan, Liechtenstein), operating in a wavelength range of 385 to 515 nm, and with an output irradiance of 1200 mW/cm^2^ (Figure 1b). While an FBG is a periodic modulation of the refractive index of the fiber core, obtained by the exposition of the fiber, for instance, to a UV laser (Section 3.2), the influence of the LED light-curing unit in the FBG′s refractive index modulation is negligible. The LED operating wavelength range is not suitable to promote the fiber core′s refractive index change, and apart from this, the two used sensors are not in direct contact with the LED (an FBG is embedded in the resin, and the other is an FBG inside a needle, also embedded into the composite). Experimental testing was repeated alternately for each resin, performing a total number of ten samples per resin (n = 10). The room temperature was set to 22 °C. Figure 1c) shows a schematic representation of the experimental setup used to assess the polymerization shrinkage of the RBCs.

### 3.4. Statistical Analysis

The statistical analysis was performed using SPSS 21.0 (SPSS Inc., Chicago, IL, USA). The data was tested for normal distribution (Shapiro-Wilk, *p* > 0.05). Two-way repeated measures ANOVA with Greenhouse-Geisser correction followed by Bonferroni´s post-hoc test was used to analyze linear shrinkage data. A 95% level of significance was adopted (α = 0.05).

## 4. Results

**Figure 2:**
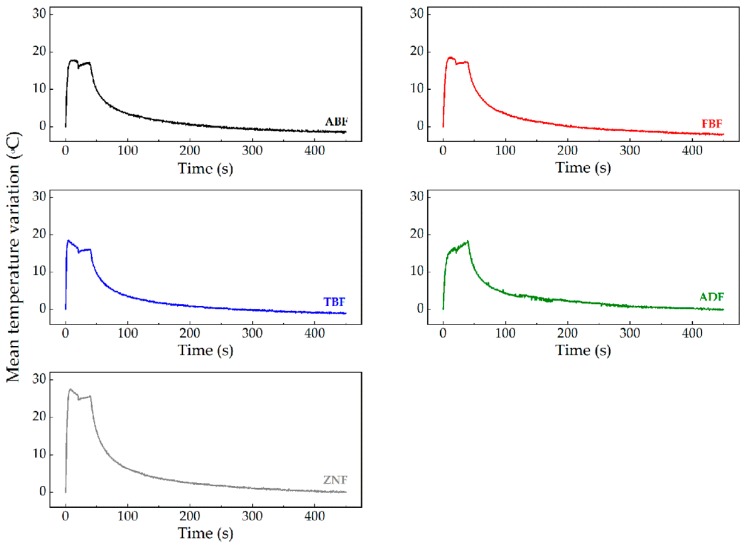
Mean temperature variation induced during the light curing of the RBCs.

**Figure 3:**
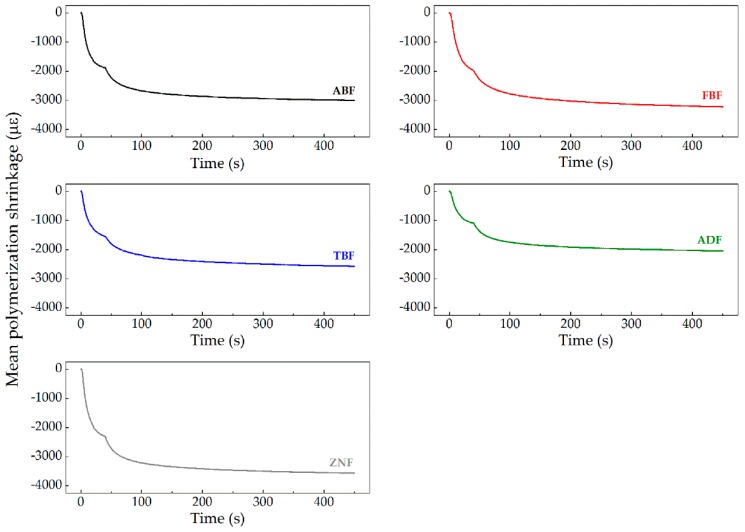
Graphic representation of the mean polymerization shrinkage of the RBCs over time.

The evolution of the temperature variation induced during the light curing of the resins is presented in Figure 2, reflecting a similar pattern for all tested composite resins. The real-time profiles of the polymerization shrinkage (expressed in microstrain) for each RBC are depicted in Figure 3. The latest data were obtained from the subtraction of the thermal effect on the values collected by the FBG in direct contact with the resins. Since the difference in the thermal sensitivity coefficient of the FBG inside and outside the needle is negligible, it is only required to subtract the Bragg wavelength shift obtained with the FBG inside the needle to the Bragg wavelength shift registered by the FBG embedded in direct contact with the RBC. After that, these data were divided by the sensitivity coefficient of 0.0012 ± 0.0001 nm/με (Section 3.2 and Equation (2)), obtaining the polymerization shrinkage expressed in microstrain. The results presented in both Figure 2 and Figure 3 are the average curves resulting from the 10 tests performed for each of the RBCs. Table 2 presents the mean polymerization shrinkage for the 5, 10, 40, 60, 150, and 300 s instants.

Two-way repeated measures ANOVA revealed different patterns of polymerization shrinkage over time for the RBCs tested (composite*time interaction, *p* < 0.0001). For all resins, the shrinkage curve was steep in the first 40 s, which coincides with the curing period, followed by a gradual increase.

The mean linear shrinkage curves as a function of the time reveal a stabilization of the recorded data at 150 s, as no significant differences of the shrinkage values were detected for any material from 150 to 300 s (*p* > 0.05). For the remaining evaluation periods, all RBCs revealed a statistical significant increase of deformation values (*p* < 0.029).

The ormocer-based bulk-fill material ADF developed significantly lower linear shrinkage, compared to all other resins, for the evaluated periods (*p* < 0.009). Inversely, the diatomita-based material ZNF showed the highest shrinkage values, statistically different from TBF and ADF (Table 2). The polymerization shrinkage of the composite specimens after light-curing decreased in the following order: ZNF > ABF > FBF > TBF > ADF (*p* < 0.05).

**Figure 4:**
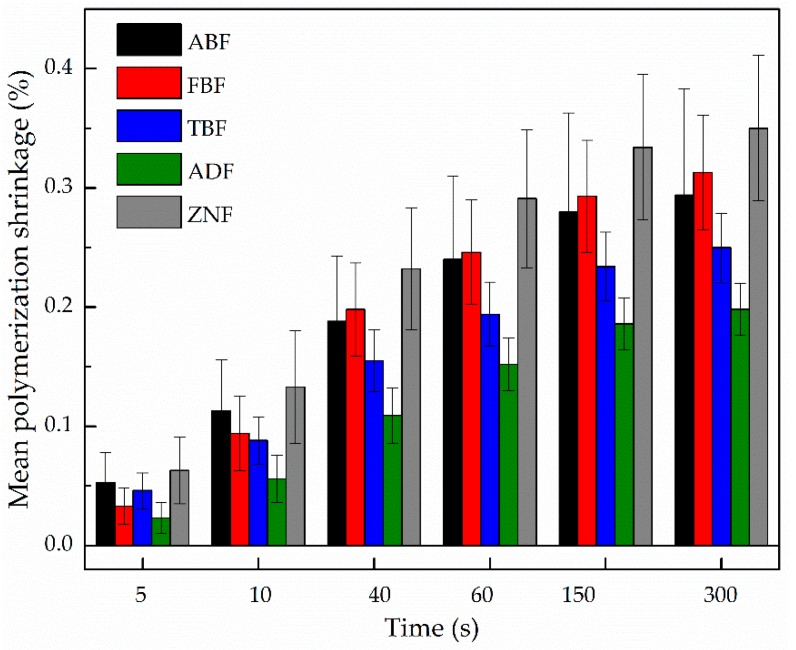
Comparison of the mean linear polymerization shrinkage (%) in different instants, for the RBCs studied.

For comparison purposes, the values of linear polymerization shrinkage obtained for the instants 5, 10, 40, 60, 150, and 300 s were expressed in percentage, according to Equation (3). The results are presented in Figure 4, expressing the lower shrinkage of ADF and the highest for ZNF.

(3) $$Linear\;polimerization\;shrinkage\;\left(\%\right)=Linear\;polimerization\;shrinkage\;\left(\mu \varepsilon \right)\;\times 10^{-6}\;\times 100$$

## 5. Discussion

In the current study, linear polymerization shrinkage of three bulk-fill base composites, a diatomite-based resin composite and a pure ormocer were compared. The results of this study showed significant differences of linear polymerization shrinkage between them, which led to the rejection of the null hypothesis. Polymerization shrinkage of composite resins results from the molecular re-arrangement of monomers, which in the pre-polymerized phase, are distanced by van der Walls forces around 0.3 Å. During polymerization, the breakage of double carbon bonds and subsequent formation of shorter simple covalent carbon-carbon bonds around 0.1 Å produces cross-linked polymer chains with an inherent resin volumetric loss [2,10].

The shrinkage leads to the deformation of the composite resins during the curing process, which, being constraint by bonding to cavity walls generates stress. However, the main factors influencing stress developments are not only related to the composite polymerization shrinkage, but also with elastic modulus development, quality of dentin adhesion, cavity configuration factor (C-factor), cavity size and compliance. Increasing stress during polymerization may overcome adhesive bond strengths, causing loss of retention and/or marginal gap formation [5,10,22,23]. Polymerization stress showed a strong correlation with post-gel and a weaker correlation with total shrinkage suggesting that, for materials with dissimilar organic and inorganic contents, differences in reaction kinetics and polymer structure affect their viscoelastic behavior and conversion reached at vitrification [4]. In the present study, the influence of the elastic modulus variation during the curing process, more properly from the point of gelation, were also accounted in the total Bragg wavelength shift. However, in future work this contribution should be discriminated from the polymerization shrinkage resulting only from the monomer to polymer conversion, as proposed by Wang et al. [3]. Again, it should be emphasized that low volumetric shrinkage does not necessarily correspond to a low polymerization stress development. Indeed, in order to effectively reduce polymerization shrinkage stress, the role of the elastic modulus must be considered [2,3,4].

Different strategies to deal with shrinkage stress have been broadly reported and discussed [5]. Modification of the resin matrix and filler phase has been declared as the major contributors for the minimization of stress development. The technology used for decreasing stress in the formulation of low-shrinkage and bulk-fill materials have shown to be a promising application for reducing and controlling stress development. Further, the use of gold-standard adhesive systems along with modified polymerization techniques allowing an extension of the pre-gel phase curing reaction of RBCs is also one important clinical approach for effective stress relief [6].

As shown in the literature, polymerization shrinkage is predominantly a resin matrix property, since it depends on the degree of conversion of monomers, reason why the composition of composite resins should be carefully analyzed. Conversely, the increase in the filler fraction incorporated in the matrix of a composite resin usually leads to a decrease of its polymerization shrinkage, since the overall matrix content is reduced [5]. For the composite resins studied, filler fraction by volume varied from 69% for ADF, 65% for ZNF and ABF, and 58.4% for FBF and 61% for TBF. Findings of the study showed that ZNF group originate the highest linear shrinkage despite its relative higher filler content. It can be considered that the matrix type and arrangement of the filler and matrix fraction in ZNF may be the cause of this result. ZNF contains high and low molecular weight monomers, including TEGDMA that may be responsible to the increase in linear polymerization shrinkage, as this monomer raises the mobility of molecules during polymerization, increasing their degree of conversion and, consequently polymerization shrinkage. In addition, co-polymerization of Bis-GMA with UDMA and TEGDMA increases conversion and creates highly cross-linked, dense and stiff polymer networks [24,25]. Besides, this new composite resin contains diatomite as filler, which presents a permeable structure of nanometric pores, contrasting with the conventional silica fillers. According to the manufacturer, this architecture allows the permeation of monomers through the pores of the diatomite particles with intrinsic improvement of the resin composite mechanical and optical properties [26]. No publications are available in the literature investigating the polymerization shrinkage of this specific resin composite. Nevertheless, other studies found an inferior mechanical behavior of ZNF when compared to other resin composites [27,28].

Bulk filling techniques are undoubtedly more user friendly than the necessary meticulous incremental layering techniques and speed up restorative procedures, reason why they are preferred by the clinicians. Only few clinical trials report the comparison of restorations with bulk-fill base and conventional composites, showing promising results for the former. Nevertheless, some of those trials do not detail information regarding cavity depth and size, or the number of increments applied in the restoration, limiting the full extrapolation of their potential positive outcomes [10]. In vitro findings report more consistently a lower shrinkage with bulk-fill materials when compared to their conventional counterparts, enhancing that polymerization shrinkage of bulk-fill RBCs are product dependent [9,11,29,30]. Our results corroborate those studies. Among the tested bulk-fill materials, TBF showed the least polymerization shrinkage. This composite contains a shrinkage stress reliever, which is a filler functionalized with silane with a lower modulus of elasticity acting as a microscopic spring lessening the forces generated during shrinkage. Besides, the pre-polymerized fillers promote a low modulus of elasticity that further contributes to the reduction of the shrinkage [11]. Viscoelasticity of polymers determines its flow capacity in the early stages of the curing reaction and, consequently, their elastic modulus development during polymerization. The relationship between the modulus of elasticity and volumetric shrinkage is the most valuable way to predict shrinkage stress. However, these properties are often inversely related and mostly depend on the filler load [10]. The full-body bulk-fill composites usually exhibit less volumetric shrinkage but higher elastic modulus than the low viscosity and flowable bulk-fill composites [29,31]. Nevertheless, several studies showed that restorative paste-like composites promote better marginal adaptation than flowable composites, establishing a high correlation between linear polymerization shrinkage, shrinkage force and the percentage ratio of the imperfect margin of bulk-fill RBCs [29,32].

The significantly lower polymerization shrinkage registered by the ormocer-based composite ADF can be assigned to a network of inorganic-organic copolymers which is denser than conventional dimethacrylate monomers. Consequently, double bonds are less reachable and reduced conversion with high amount of unreacted double bonds may be expected [16,17]. Although this may contribute to the reduction of linear shrinkage and shrinkage force, other studies showed that this composite is more susceptible to mechanical degradation induced by water, which eventually translates in a reduction of its mechanical behavior when compared to conventional Bis-GMA-containing resin composites [17,33].

Many scientific papers published have focused on different approaches to assess composite polymerization shrinkage. However, the heterogeneity of the experimental setups along with the fact that shrinkage values significantly depends on the method used to measure it, limits direct comparisons between reported results [10]. The use of the FBG based technology for measuring the polymerization shrinkage in a real-time recording modus has been described in previous publications, revealing results consistent with the present study, although different composite resins were evaluated [20,34,35]. Linear polymerization shrinkage of 0.32% for the hybrid composite resin Filtek Z250 and 0.15% for Z100, assessed with FBG sensors, were pointed out by Anttila et al. [35] and Milczewski et al. [34], respectively. Rajan et al. [20] studied six different resin composites, exhibiting linear polymerization shrinkage within a range of 0.4% to 1.2%, enabling the influence of filler properties and fiber reinforcement on their results.

Some drawbacks can be pointed out to the methodology employed in this study, concerning the FBG sensors. Special caution should be attained to the cross-sensitivity to both strain and temperature, which requires specific techniques to compensate temperature, particularly, by measuring the temperature individually in order to compensate its effect on the strain values [36]. Other disadvantage consists on the inherent fragility of the fiber, which difficult the placement of the composite around it without damage to the fiber, particularly in the case of non-flowable composites. Apparently, composite resins adhere relatively easily to the fiber′s surface without requiring any pre-treatment. However, there is a lack of research data concerning the effect of some surface fiber pre-treatment on hypothetical interfacial link between the composite resin and the fiber. Despite being slightly more expensive, the optical fiber technology can provide a simple and reliable method of measuring multiple physical properties of dental composites with the same sample [20]. Therefore, the use of this methodology should be encouraged.

## 6. Conclusions

Linear polymerization shrinkage of RBCs was evaluated in real time using optical fiber Bragg grating sensors embedded in the material evidencing good reliability. Within its limitations, this in vitro study showed that Admira Fusion, an Ormocer, presented the lowest linear shrinkage over all other RBCs, while restorative bulk-fill composites exhibited an intermediate behavior. The diatomite-based RBC Zirconfill developed the highest shrinkage values.

## Figures and Tables

**Table 1:** Resin-based composites specifications.

Resin (Text Code)	Name	Manufacturer	Type	LOT n°/Shade	Composition	Filler (wt%/vol%)
ABF	Aura Bulk Fill	SDI, Victoria, Australia	Full-body bulk-fill composite	150330/BKF	Matrix: Bis-GMA, UDMA Filler: Silica, barium glass silanized	-/65
FBF	Filtek^TM^ Bulk Fill Posterior	3M ESPE, St Paul, MN, USA	Full-body bulk-fill composite	N685666/A2	Matrix: Proprietary AUDMA and AFM, UDMA, DDDMA Filler: non-agglomerated/non-aggregated 20 nm silica filler, non-agglomerated/non-aggregated 4- to 11-nm zirconia filler, aggregated zirconia/silica cluster filler (comprised of 20-nm silica and 4- to 11-nm zirconia particles), ytterbium trifluoride filler consisting of agglomerate 100-nm particles	76.5/58.4
TBF	Tetric^®^ N-Ceram Bulk Fill	Ivoclar Vivadent AG, Schaan, Liechtenstein	Full-body bulk-fill composite	U03089/IVA	Matrix: Bis-GMA, Bis-EMA, UDMA, additives, initiators, stabilizers, pigments Filler: Barium alumino silicate glass, prepolymer (isofillers), ytterbium	78/61
ADF	Admira Fusion-Ormocer^®^	VOCO, Cuxhaven, Germany	Nano-hybrid Ormocer-based composite	1635576/A2	Matrix: Ormocer (organically modified silicic acid) Filler: Barium-aluminum-glass, pyrogenic silicon dioxide	84/69
ZNF	Zirconfill^®^	Technew, Rio de Janeiro, Brazil	Full-body composite	16004/A2D	Matrix: Bis-GMA, Bis-EMA, TEGDMA, UDMA Filler: Silica/Zirconia mixed oxide in form of nanoclusters, diatomite, barium glass	80/65

Abbreviations: Bis-GMA: bisphenol A glycidyl methacrylate; bisEMA: ethoxylatedbis-phenol A dimethacrylate; bisEMA(6): (2,2-bis[4-methacryloxypolyethoxyphenyl)propane]; DMA: dimethacrylate; UDMA: urethane dimethacrylate; TEGDMA: triethylene glycol dimethacrylate; DDDMA: 1,12-dodecanediol dimethacrylate; proprietary AUDMA: high molecular weight aromatic dimethacrylate; proprietary AFM: addition-fragmentation monomers.

**Table 2:** Mean (standard deviation) of the polymerization shrinkage (με).

Resin	Time												Time′s Effect *p*
	5 s		10 s		40 s		60 s		150 s		300 s		
ABF	−534.47 (250.74)	ab	−1131.57 (428.90)	ab	−1882.62 (550.39)	ab	−2396.12 (700.81)	ab	−2797.71 (829.36)	ab	−2938.42 (889.22	ab	<0.01
FBF	−334.13 (152.69)	ac	−935.43 (307.67)	abc	−1977.63 (385.49)	ab	−2456.97 (440.32)	ab	−2932.94 (470.41)	ab	−3131.14 (484.17)	ab	<0.01
TBF	−459.24 (154.74)	abc	−880.74 (202.31)	abc	−1547.67 (225.77)	ac	−1940.65 (274.02)	ac	−2337.71 (292.04)	ac	−2495.34 (293.26)	ac	<0.01
ADF	−228.36 (126.10)	c	−555.50 (204.86)	c	−1091.59 (233.36)	c	−1520.90 (221.22)	c	−1855.50 (218.35)	c	−1980.66 (224.60)	c	<0.01
ZNF	−631.40 (281.55)	b	−1328.11 (469.34)	b	−2315.22 (505.92)	b	−2913.60 (580.52)	b	−3343.62 (614.39)	b	−3497.33 (611.35)	b	<0.01
Resin′s effect *p*	<0.01		<0.01		<0.01		<0.01		<0.01		<0.01		

Similar lowercase within the same period of evaluation indicate RBCs that do not differ statistically (*p* > 0.05).
